# Oculocutaneous Albinism and Squamous Cell Carcinoma of the Skin of the Head and Neck in Sub-Saharan Africa

**DOI:** 10.1155/2015/167847

**Published:** 2015-08-12

**Authors:** P. T. Lekalakala, R. A. G. Khammissa, B. Kramer, O. A. Ayo-Yusuf, J. Lemmer, L. Feller

**Affiliations:** ^1^Department of Maxillofacial and Oral Surgery, Sefako Makgatho Health Sciences University, Pretoria 0204, South Africa; ^2^Department of Periodontology and Oral Medicine, Sefako Makgatho Health Sciences University, Pretoria 0204, South Africa; ^3^School of Anatomical Sciences, Faculty of Health Sciences, University of the Witwatersrand, Johannesburg 2000, South Africa; ^4^School of Oral Health Sciences, Sefako Makgatho Health Sciences University, Pretoria 0204, South Africa

## Abstract

Oculocutaneous albinism which is characterised by impaired melanin biosynthesis is the most common inherited pigmentary disorder of the skin and it is common among Blacks in sub-Saharan Africa. All albinos are at great risk of developing squamous cell carcinoma of sun-exposed skin, and Black albinos in sub-Saharan Africa are at about a 1000-fold higher risk of developing squamous cell carcinoma of the skin than the general population. In Black albinos, skin carcinoma tends to run an aggressive course and is likely to recur after treatment, very probably because the aetiology and predisposing factors have not changed. Prevention or reduction of occurrence of squamous cell carcinoma of the skin in Black albinos might be achieved through educating the population to increase awareness of the harmful effects of exposure to sunlight and at the same time making available effective screening programs for early detection of premalignant and malignant skin lesions in schools and communities and for early treatment.

## 1. Introduction

Skin pigmentation varies between persons and is determined by multiple factors including the number and the metabolic activity of the melanocytes in the basal cell layer of the epidermis, the melanogenic activity of the melanosomes within these melanocytes, and differences in number, size, and distribution of the melanosomes. Differences in the type of melanins and differences in the degree of arborisation of the dendritic processes of the melanocytes and in the transfer of melanosomes from these processes to surrounding keratinocytes will also affect the pigmentation of the skin [1, 2].

Melanin biosynthesis is regulated by several factors, particularly by melanocortin-1 receptor (MC1R) on the melanocytes and its ligand, *α*-melanocyte stimulating hormone (*α*MSH). Cytokines and growth factors in the microenvironment and the degree of basal activity of tyrosinase, tyrosinase related protein 1 (TRP1), and membrane associated transport proteins are additional factors regulating this biosynthesis [2, 3]. One of the important functions of melanin is protecting the skin and eyes from the harmful effects of ultraviolet radiation (UVR).

Melanocytes of the skin and uveal tract of the eyes are derived from neural crest cells. A number of genes control the proliferation and differentiation of neural crest cells and also regulate the migration of precursor melanocytes to their ultimate positions in the skin and eye. Microphthalmia transcription factor (MITF) is the master regulator of melanocyte development, function, and survival [4] and is responsible for modulating expression of some melanocyte-specific proteins [5]. Following differentiation of melanocytes, MITF regulates expression of genes during exposure to UVR, thus assisting in tanning of the skin [6]. Transfer of the melanin to surrounding keratinocytes and the production of a nuclear cap protect the DNA from UVR damage [2, 7]. The degree of pigmentation of the skin is said to correlate inversely with the risk of sun-induced skin cancers [6].

Oculocutaneous albinism (OCA) is an autosomal recessive disorder of melanocyte differentiation brought about by defects in the pathway of melanin biosynthesis, by defects in melanosome biogenesis or function, or by dysregulation of intracellular transport and localization of proteins essential for melanin production [8]. The typical clinical manifestations of OCA can vary greatly and comprise partial or complete lack of melanin pigmentation of the skin, reduced visual acuity, and ocular nystagmus [8–10]. The number and distribution of melanocytes in OCA are normal. Albinos with total lack of melanin have white skin and hair and pink eyes, and they sunburn easily [11]. However, albinos with only partially reduced capacity for melanin biosynthesis will acquire some pigmentation during life [12].

OCA predisposes to squamous cell carcinoma of the skin (SCCS), particularly of the sun-exposed head and neck [13, 14]. SCCS is more frequent, runs a more aggressive course, and tends to have a higher rate of recurrence in Black albinos than in normally pigmented persons, whether Black or White [8, 15–17]. Surprisingly, in Black albinos, SCCS of the head and neck is more prevalent than basal cell carcinoma, and cutaneous melanoma of the head and neck is rare [18, 19]. In this short paper we will discuss some pathogenic mechanisms of SCCS in albinos and elaborate on public health measures to reduce its incidence.

## 2. Oculocutaneous Albinism

There are five types of OCA; of these OCA1 and OCA2 are by far the most frequent types. OCA type 1 (OCA1) occurs with a frequency of about 1/40 000 worldwide [20], affects different racial/ethnic groups equally, and is characterized by loss of function of the enzyme tyrosinase (TYR) as a result of a mutation in the TYR gene. Tyrosinase is the critical enzyme in the biosynthesis of both brown-black eumelanin and yellow-red pheomelanin. Persons with OCA1A have completely nonfunctional TYR, with no melanin production, while in persons with OCA1B there is some tyrosinase functional activity with limited melanin production [10, 12, 21].

OCA2 is the most common form of albinism worldwide [6], prevalent in southern Africa. It affects Blacks more commonly than Whites and is characterized by mutations in the OCA2 gene (formerly known as the P gene) that encodes the p protein [10, 21]. Its precise functions are not fully understood but p protein appears to be involved in transporting proteins to the melanosome, in stabilizing the melanosomal protein complex, and in regulating melanosomal pH and/or glutathione metabolism, all of which are important to melanin production [10, 21, 22]. Albinos with an OCA2 phenotype have no eumelanin but have some pheomelanin which may increase with age [9, 10, 23]. OCA2 is the most common phenotype affecting Black South African albinos, with an overall prevalence of OCA2 albinism of one in 3900 persons [23–25].

The OCA3 and the OCA4 phenotypes of albinism are caused by mutations in genes encoding tyrosinase related protein 1 (TRP1) and membrane associated transport protein (MATP), respectively [9]. TYPR1 is an enzyme which stabilizes tyrosinase. Mutations in TYPR1 are associated with early degradation of tyrosinase and with delayed maturation of melanosomes [16, 21]. MATP functions as a melanosomal membrane transporter of proteins necessary for melanin biosynthesis, and mutations in the MATP gene consequently cause hypopigmentation and the OCA4 phenotype of albinism [16, 21]. OCA5 phenotype is linked to an as yet unidentified specific gene mapped to the 4q24 chromosomal region and was discovered in the members of a consanguineous Pakistani family [26, 27].

At birth, persons with the different phenotypic forms of OCA all have white hair and very pale and pink-white skin. Those with OCA1B, OCA2, OCA3, or OCA4 will acquire some pigmentation during life, but those with OCA1A will remain completely unpigmented [9, 21]. The degree of pigmentation associated with OCA5 phenotype is not clear [26, 27].

## 3. Melanin and SCCS

Cutaneous melanin, particularly brown/black eumelanin, provides protection both against sunlight and against oxidative stress-induced DNA damage so that dark-skinned persons have a lower frequency of SCCS than do light-skinned persons. However, the photoprotection afforded by melanin is not complete even in dark-skinned persons who can also sustain sunlight-induced DNA damage, but this damage is usually of a degree that can be repaired by cellular DNA repair mechanisms thus reducing the risk of malignant transformation. On the other hand, in light-skinned persons who lack sufficient melanin to provide effective protection against sunlight, the extent of the sunlight-induced DNA damage may exceed the capacity of these cellular DNA repair mechanisms, with increased risk of malignant transformation [12]. Albinos who have very little, if any, melanin in their skin are thus very susceptible to sunlight-induced SCCS.

In this connection it may be noted that xeroderma pigmentosum, in which there is inherently impaired functional activity of DNA repair mechanisms, can affect either dark-skinned or light-skinned persons, and both groups have equal frequency of SCCS [28]. This highlights the fact that effectively functioning cellular DNA repair mechanisms are more important in preventing SCCS than the quantum of melanin pigment in sun-exposed skin.

The melanin present in OCA is mainly pheomelanin, while the production of eumelanin is minimal [29]. Eumelanin has an important photoprotective role, but although pheomelanin does afford some sunlight photoprotection, during its biosynthesis, reactive oxygen species (ROS) which are carcinogenic are generated. Therefore, in albinos, both reduction in eumelanin photoprotection and elevation of pheomelanin-derived ROS are implicated in SCCS [9].

The biosynthesis of both types of melanin, brown/black eumelanin and yellow/red pheomelanin, is controlled to a large extent by the melanocortin-1 receptor (MC1R) on the melanocytes. Albinos with the OCA2 phenotype who possess polymorphic gene variants of MC1R, in contrast to most other albinos, may have reddish hair and a yellowish tinge to their skin [30]. In this regard, it has been reported that the OCA2 phenotype of albinism can be brought about solely by a mutated MC1R gene [31].

The activity of some MC1R variants can counteract apoptosis and reduce the capacity for the DNA repair of melanocytes. They can also indirectly reduce protection of keratinocytes from sunlight-induced DNA damage because of the reduced eumelanin production, thus increasing the risk of skin cancer. MC1R gene variants are associated not only with dysregulated melanin production and reduced tanning capacity, but also with modulation of host immunoinflammatory responses which are important in immune surveillance and in the killing of sunlight transformed keratinocytes [1, 29, 32]. In this regard, genetic polymorphisms of other genes encoding agents involved in melanin biosynthesis (TYR and TRP1), beside determining skin pigmentation, are in fact also factors contributing to the risk of developing skin cancer [1]; and it has been suggested that functionally active tyrosinase has the capacity to protect against oxidative DNA damage [33].

## 4. Sunlight-Induced Malignant Transformation

As most persons with severe forms of OCA are very prone to sunburn [21], the progenitor basal cell keratinocytes of sun-exposed skin of albinos are at great risk of undergoing sunlight-induced malignant transformation. SCCS in albinos can arise* de novo* or from premalignant actinic lesions such as sunlight keratosis, in which the keratinocytes have already undergone sunlight-induced initial transformation. The basal cell keratinocytes will sustain DNA damage of different degrees of severity according to the intensity and duration of exposure to sunlight. Normally, the p53 tumour-suppressor gene arrests the cell cycle, allowing for the repair of the damaged DNA, or promotes apoptosis if the DNA damage is irreparable. However, if sunlight induces mutations in p53 itself rendering it dysfunctional, there will be propagation of damaged DNA by cell division, resulting in a precancerized epithelial field composed of a clone of initially transformed keratinocytes with genomic instability. This genomic instability predisposes the initially transformed keratinocytes to additional genetic alterations and may drive the processes of clonal divergence with consequent clonal expansion of keratinocytes possessing a selective growth advantage, ultimately giving rise to a frank SCCS [12, 34, 35]. The risk of SCCS is proportional to the accumulated quantum of UVR absorbed by the keratinocytes [31], but ultimately the potential for malignant change is determined by the number of genetic insults. Thus, numerous smaller frequent exposures to sunlight are more likely to be carcinogenic than greater but infrequent exposures to sunlight [36], because each exposure event has the potential to cause a genetic change. The more the genetic alterations occur, the greater the chance of malignant transformation will be.

As albinos are photosensitive and tend to sunburn readily, sunlight-induced local inflammation in the skin can be an additional factor in bringing about an increase in proliferation and longevity of basal keratinocytes favouring initial malignant transformation. After sunburn, local inflammatory cell-derived ROS can directly cause DNA damage, dysregulating the mechanisms not only of DNA repair and of cell cycle checkpoint control but also of apoptosis, promoting evolution of SCCS [37].

At the molecular level, UVR-induced DNA damage is characterized by substitution of specific nucleotides, particularly C > T and CC > TT transitions found in the p53 gene that encodes the p53 protein, which normally regulates the cell cycle, apoptosis, and DNA repair. Sunlight regularly causes these genetic alterations that are referred to as UVR-associated “signature mutations,” and these signature mutations drive the malignant transformation of sunlight-induced SCCS [36, 38].

Initially transformed keratinocytes are immunogenic and thus generate immune responses which can modulate or control tumourigenesis; but sunlight-induced immunosuppression may critically interfere with this protective mechanism [39].

**Figure 1:**
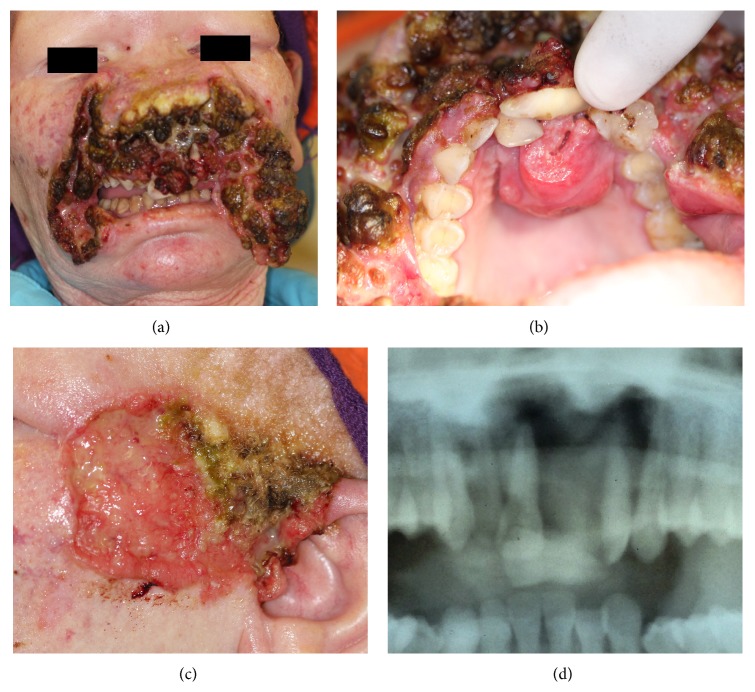
Squamous cell carcinomas (SCCs) in a 38-year-old HIV-seropositive Black albino woman. (a) SCC of the nose, cheeks and the lips, and (b) the labial mucosa and anterior part of the palate. The disease started as a small ulcer on the upper lip 18 months previously, progressing rapidly to involve the mouth and to destroy the lower face. (c) SCC of the left ear and temple started 8 months previously. (d) Panoramic radiograph showing destruction of the anterior maxilla. Histopathologically, the carcinoma was poorly differentiated. The patient died before investigation for metastases could be done. The extensive and striking sunlight-induced malignant facial damage is tragic evidence of the consequence of lack of sun-protection from an early age and of appropriate early medical care.

The risk of SCCS in Black albinos is 1000 times greater than the risk in the general population, and the head and neck region is most frequently affected (Figure 1) [14, 40]. By the third decade of life, many Black albinos in Africa will have developed potentially fatal SCCS [16, 40], but if diagnosed at an early stage, SCCS is curable by surgical excision. Timely recognition of the disease is therefore crucial.

**Figure 2:**
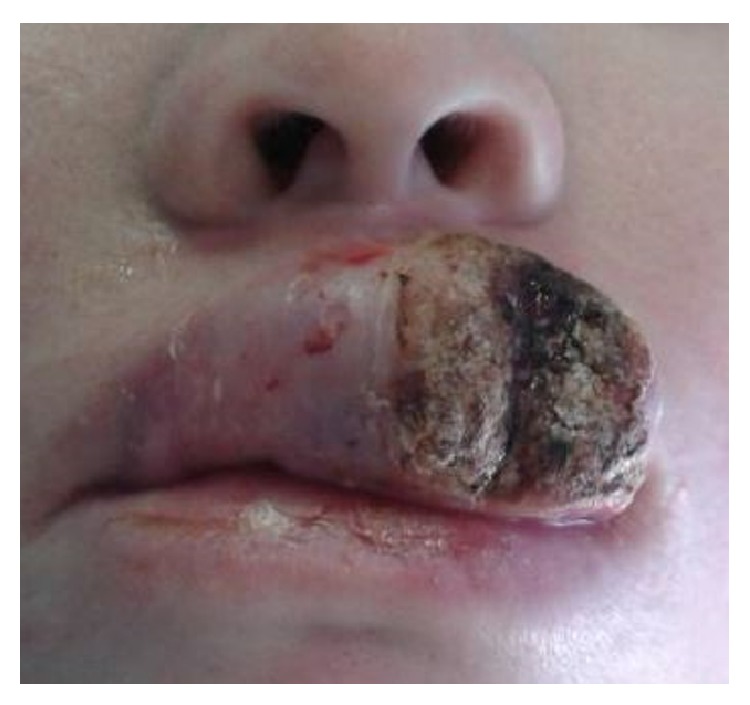
A 21-year-old HIV-seropositive Black albino woman with an exophytic crusted SCC of the upper lip, twelve months after she first noticed a small painless erosion. There was neither local lymph node involvement nor distant metastasis, and microscopically the carcinoma was moderately differentiated.

It is not clear what effect HIV-induced immune impairment or the virus itself may have on the aetiopathogenesis of SCCS [41, 42]. However, the frequency of squamous cell carcinoma of the lip (Figure 2) is reportedly increased in HIV-seropositive immunosuppressed subjects as compared to immunocompetent HIV-seronegative subjects [42, 43]. What the impact of HIV or HIV-induced immune impairment on SCCS in albinos is, is unknown and requires further research [42, 44].

## 5. Public Health Measures to Minimise SCCS in Albinos

The objectives of cancer management in the field of public health are reduction of the incidence, early detection, and prompt treatment of the disease when it occurs. Universal precautions against sunlight exposure should be introduced early in childhood, continue throughout life, and should include minimising of outdoor activities during peak sunlight hours, the wearing of protective clothing to cover as much of the skin as possible, and the use of sunscreen preparations for exposed skin [12, 34].

However in general, public health measures to minimise sunlight-induced damage to the skin of populations in Africa are often unsuccessful because of poverty, lack of understanding of the problem, and lack of compliance even when they have been informed about preventive measures [13, 40]. Moreover, in Africa, Black albinos are often subject to social discrimination because of superstitious beliefs and the stigma associated with albinism [12, 45, 46]. They are therefore often shunned by their communities with consequent delay in seeking and obtaining medical treatment until late in the course of any premalignant or malignant actinic lesions. Thus, by the time of diagnosis, SCCS in Black albinos is often advanced and has a poor prognosis [12, 47]. It has been reported that on average Black albinos in Africa seek medical treatment 9–12 months after the onset of any actinic lesions [40, 47].

Sadly, about 40% of Black albinos in Africa with SCCS do not complete their treatment owing either to financial constraints [13] or to distance from medical facilities and are lost to follow-up. These may be the reasons why SCCS are more frequent and tend to have a higher rate of recurrence in Black albinos in Africa than in normally pigmented persons, whether Black or White [8, 15, 16].

It may be possible to reduce the prevalence of SCCS in Black albinos in Africa [47] if public health personnel were regularly to visit remote villages to screen for premalignant and malignant skin lesions in the albino population and to educate them on the harmful effects of exposure to solar radiation. This would need to be supplemented by accessible treatment centres, where treatment of early SCCS could be done [48], and by the establishment of educational support groups.

## 6. Conclusion

While there is still a need for further research on prevalence of albinism in Africa, measures directed at reducing the incidence of SCCS in members of the albino community and at alerting those with premalignant actinic skin lesions to the benefits of early detection and treatment should include education about the risk factors associated with SCCS and about the hazards of delaying the seeking of professional advice. There is also a need to educate the population at large about albinism with the aim of promoting greater social integration of albinos into their communities.

Professional measures to prevent and control SCCS in albinos should include the institution of screening programmes with a view to identifying potentially malignant actinic skin lesions and detection of early SCCS and to make available immediate effective psychological and medical treatment.

## Abbreviations

MC1R: Melanocortin-1 receptor
*α*MSH: *α*-melanocyte stimulating hormone
TRP1: Tyrosinase related protein
UVR: Ultraviolet radiation
MITF: Microphthalmia transcription factor
OCA: Oculocutaneous albinism
SCCS: Squamous cell carcinoma of the skin
TYR: Tyrosinase
MATP: Membrane associated transport protein
ROS: Reactive oxygen species.
